# Asymmetric Extraction Treatment in a Middle-Aged Patient with Dental Crowding and Protrusion using Clear Aligners

**DOI:** 10.1155/2023/8836409

**Published:** 2023-08-30

**Authors:** Xiujin Xing, Hanglin Qin, Jing Sun, Kun Li

**Affiliations:** ^1^Department of Orthodontics, Yantai Stomatological Hospital Affiliated to Binzhou Medical College, Yantai, China; ^2^Wolong Division, Yantai Stomatological Hospital Affiliated to Binzhou Medical College, Yantai, China; ^3^Fushan Division, Yantai Stomatological Hospital Affiliated to Binzhou Medical College, Yantai, China

## Abstract

Frequently, orthodontic treatment involves symmetrically extracting premolars to correct severe crowding or protrusion. Nevertheless, in some cases, a more reasonable alternative may be to remove teeth with poor prognoses to improve protrusion and relieve crowding. A middle-aged woman sought treatment for dental protrusion and crowding. Her mandibular right first molar had been treated with root canal therapy due to pulpitis, but she still felt uncomfortable. In addition, her maxillary left second premolar had become carious. Extractions of the maxillary right first premolar and left second premolar, as well as mandibular right first molar and left first premolar were chosen to resolve the occlusion problems. The patient opted for clear aligners on the demands of esthetics as well as comfort. Following orthodontic treatment, the patient attained properly aligned teeth, a pleasing smile, and a facial profile that exhibited greater harmony. This case report demonstrates that, under proper planning, clear aligners are capable of handling challenging cases, including those involving middle-aged individuals and molar extractions.

## 1. Introduction

Dental crowding and protrusion are the most common malocclusions in the Asian population, and symmetric extraction of premolars and space closure are appropriate solutions. However, when the patient seeking treatment for severe crowding or protrusion has hopeless teeth other than premolars, an unusual treatment pattern of extracting these teeth can be preferentially considered.

The ongoing search for innovation in orthodontic treatment has boosted the evolution of clear aligners to offer patients more comfort, shorter treatment time, improved posttreatment stability, and fewer side effects [1]. Although initially for relatively simple cases, the scope of clear aligner technology in orthodontics has expanded to more complex malocclusions in recent years, such as severe protrusion. Nevertheless, because the material of clear aligners are not rigid enough to retain the original shape, their applications in tooth extraction cases are challenging. This can cause the mesial tipping of adjacent teeth toward the extraction space, leading to non-parallelism of roots, reduction of extraction space and anchorage loss, and eventually increases the difficulties in the treatment [2–4]. In other words, the virtual simulation of clear aligner treatment is more a process of changes in the shapes of clear aligners than predicting the final therapeutic effects. Thus, adequate perception of the properties of clear aligners and reasonable design for tooth movements are essential.

This case report portrays an asymmetric extraction treatment in a middle-aged patient using clear aligners. To correct the existing malocclusion characterized by dental protrusion and crowding, the patient was managed with extractions of three premolars and one hopeless first molar.

## 2. Case Presentation

The patient was a 46-year-old woman, with the chief complaint of protrusive incisors and lips. She was particularly concerned about the esthetic impairment during orthodontic treatment and strongly requested invisible appliances. No systemic disease was reported.

**Figure 1:**
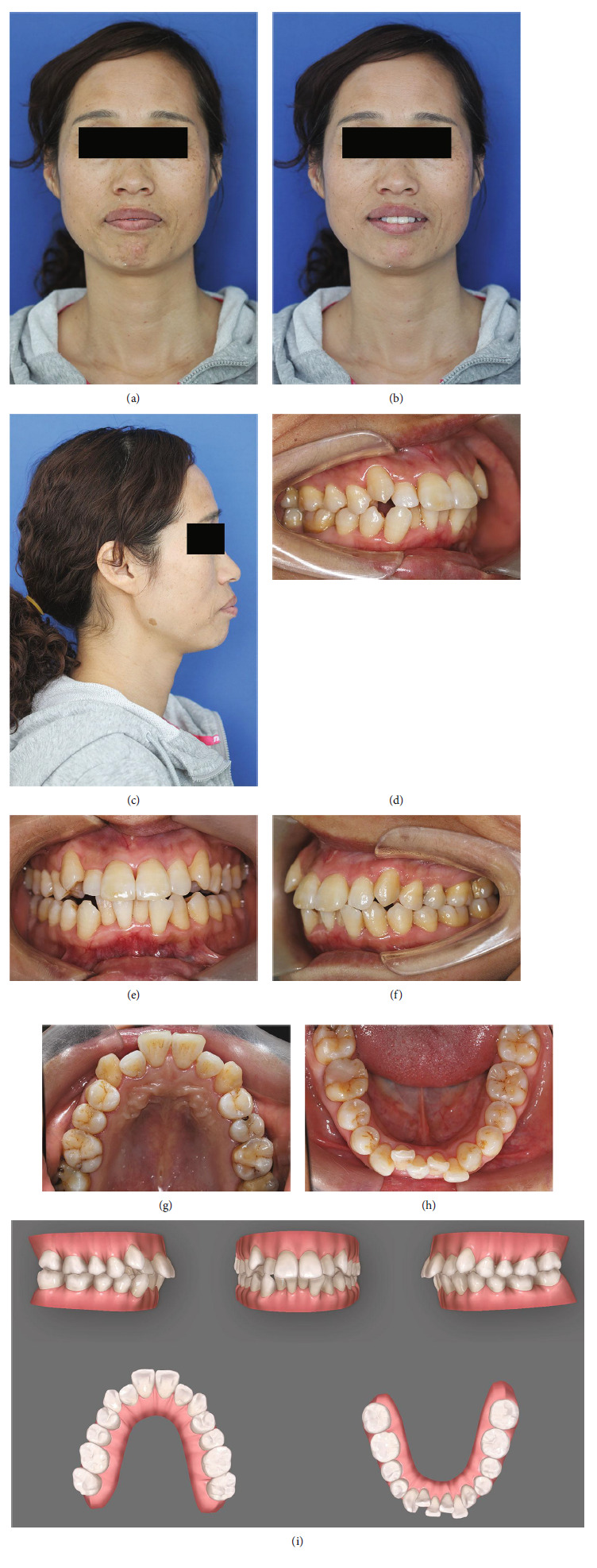
Pretreatment facial and intraoral photographs and dental casts. (a) Frontal view. (b) Smile. (c) Lateral view. (d) Right molar relationship. (e) Centric occlusion. (f) Left molar relationship. (g) Mirror view (maxillary). (h) Mirror view (mandibular). (i) Pretreatment dental casts.

Under extraoral examination (Figures 1(a), 1(b), and 1(c)), the patient exhibited a convex facial profile along with a reduced nasolabial angle. Additionally, there was a noticeable strain on her circumoral muscle when closing her mouth. There was a slight right deviation of the chin, and the right half of the face appeared larger compared with the left. Intraoral examination (Figures 1(d), 1(e), 1(f), 1(g), 1(h), and 1(i)) demonstrated Class I molar relationship and Class II canine relationship on both sides, with an overjet of 5.9 mm and an overbite of 4.0 mm. Moderate crowding as well as a deep curve of Spee could be noted. The Bolton ratio of anterior teeth was 79.0%. A large area of filling material in the mandibular right first molar and caries in the maxillary left second premolar could be observed. The periodontal status was not very fine with multifocal gingival inflammation, characterized by erythema, edema, and bleeding on probing. Probing depths ranging from 2 to 6 mm were detected with attachment loss measuring 2–5 mm, indicating the presence of periodontal pockets. Extensive gingival recession was noted, and grade III furcation involvement was detected in the mandibular right first molar.

**Figure 2:**
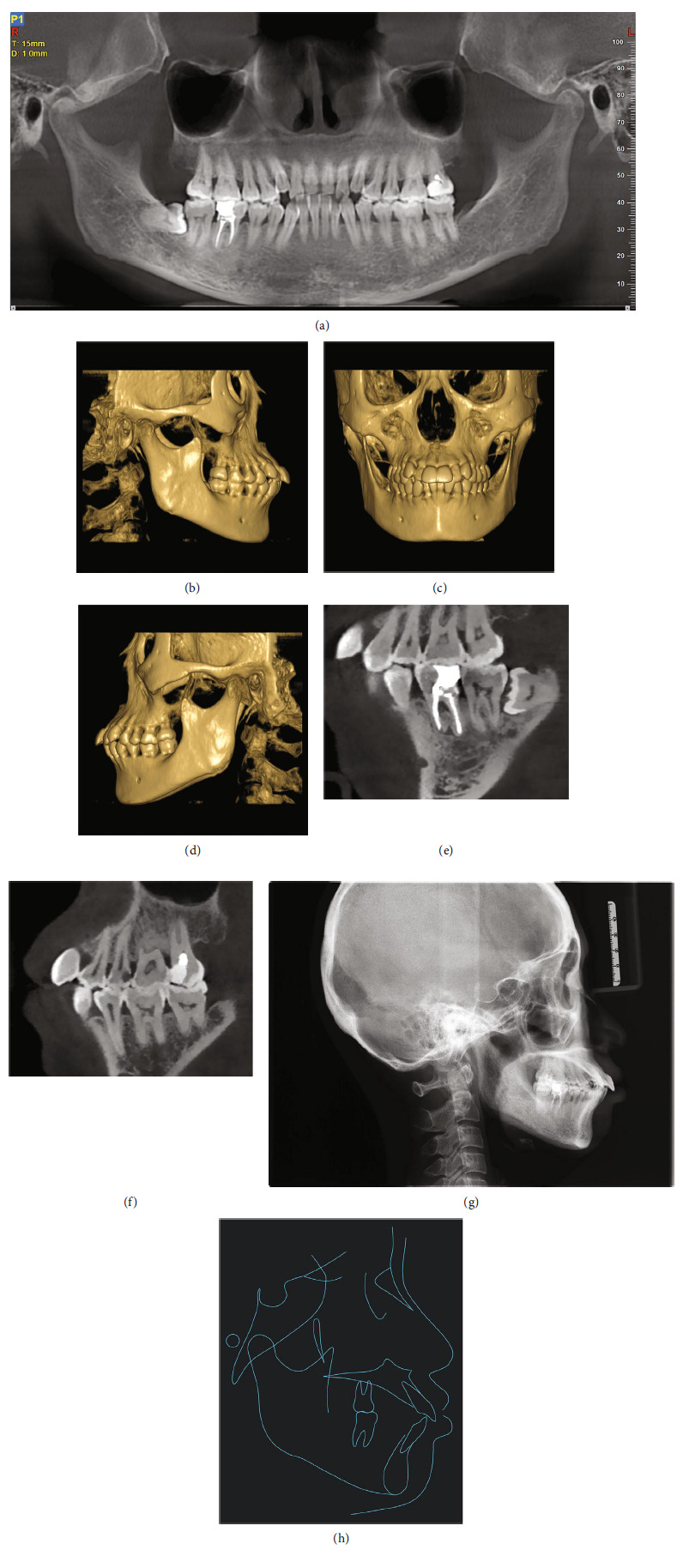
Pretreatment radiographs. (a) Panoramic radiograph sectioned from CBCT. (b–d) Three-dimensional reconstruction of CBCT. (e) The mandibular right first molar exhibited obvious radiolucency in the furcation and apical region, with root resorption. (f) The maxillary left second premolar exhibited a radiolucency in the distal regions of the crown. (g) Lateral cephalogram. (h) Lateral cephalometric tracing.

In the initial cone-beam computed tomography (CBCT; Figures 2(a), 2(b), 2(c), 2(d), 2(e), and 2(f)), horizontal and vertical resorptions of the alveolar bone was visible. Interdental bone loss was evident, with vertical and horizontal alveolar defects observed between teeth. Radiographic evidence of furcation involvement in the mandibular right first molar was also identified. The maxillary third molars and mandibular left third molar were missing. All other teeth were present but the mandibular right third molar was horizontally impacted. The mandibular right first molar had been treated with root canal therapy several years previously due to severe caries but still exhibited obvious radiolucency in the furcation and apical region, and root resorption was observed. The patient also complained that discomfort and pain had often occurred in this area during mastication. The maxillary left second premolar exhibited a radiolucency in the distal regions of the crown.

A skeletal Class I relationship was confirmed by the lateral cephalometric analysis (Figures 2(g), 2(h); Table 1). The maxilla and mandible were normally placed with an average mandibular plane angle. Both the maxillary and mandibular incisors were proclined, resulting in the lips extending beyond the E-line.

A diagnosis of skeletal Class I malocclusion with a convex facial profile, dental crowding, and protrusive incisors was made.

The treatment objectives included alleviating crowding and retracting anterior teeth to achieve optimal occlusion and improve the facial profile. Extractions were required to meet these therapeutic goals.

Before initiating orthodontic treatment, a comprehensive evaluation of the patient's condition and a thorough discussion was conducted. Multiple approaches were explored to improve dental and facial esthetics, taking into account the patient's chief complaint about the malocclusion.

Removal of four first premolars might have been the best course for treatment if all teeth were healthy. However, the mandibular right first molar with a large area of filling material exhibited images of root resorption, and radiolucency in the furcation and apical region could be observed. After consultation and discussion, even if this tooth had been successfully performed with endodontic retreatment, an unfavorable prognosis might have been its eventual downfall. Therefore, the mandibular right first molar was at risk of future extraction and implant restoration. The patient refused this option of symmetric extraction of four first premolars.

The second option was to extract the mandibular right first molar and the first premolars in the other three quadrants. Nevertheless, if this option was selected, the maxillary left second premolar should have been treated with endodontic therapy due to severe caries before orthodontic treatment.

The third option was to extract the maxillary right first premolar and left second premolar and mandibular right first molar and left first premolar. Due to the unusual extraction pattern and the large amount of space after the first molar extraction, the patient was informed that the treatment time might be extended accordingly. After being presented with all the benefits and drawbacks, the patient chose this treatment plan.

**Figure 3:**
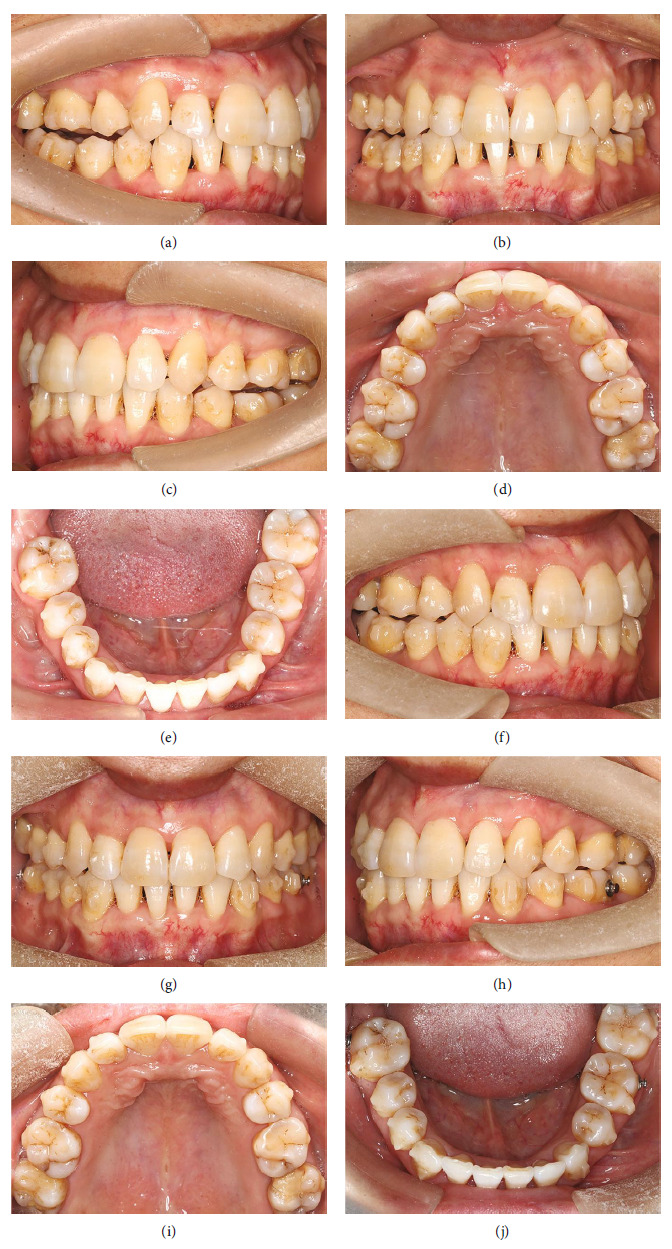
Progress intraoral photographs. (a–e) Aligner stage 62, after 22 months of treatment. (f–j). Stage 15 of the second refinement, after 35 months of treatment.

Before the orthodontic treatment was initiated, complete periodontal treatment, including scaling and root planning of all but the teeth that needed to be extracted had been performed. Meanwhile, oral hygiene instructions were provided to the patient. The indications for initiating orthodontic treatment, included proper infection control, a full-mouth plaque index within 25%, a percentage of positive bleeding on probing sites less than 30%, and no residual pockets deeper than 5 mm. In the first phase of orthodontic treatment, 62 stages of aligners were designed, and the patient was instructed to change aligners every 10 days and to wear each for 22 hours per day. At stage 62 (Figures 3(a), 3(b), 3(c), 3(d), and 3(e)), all teeth had been aligned, with all the spaces completely closed. With the anterior teeth being retracted, the facial profile and smiling esthetics had been substantially improved. The overbite and overjet also gradually improved throughout this time. However, a large range of tooth movements had caused a disordered curve of Spee with loss of incisor torque, the distal tipping of canines, and the mesial tipping of molars. The first phase ended up with a bilateral posterior open bite.

Consequently, the first refinement was initiated and continued for about 8 months using 24 stages of aligners. This phase aimed to correct the torque of the incisors, upright canines, and molars, and close the open bite, along with Class II elastics from maxillary canines to mandibular molars to settle the occlusion. After that, the posterior open bite showed gradual improvements but was still present, without any improper aligner fitting.

The second refinement was started with 15 stages of aligners aiming at leveling the curve of Spee by intruding anterior teeth and extruding posterior teeth, and this phase lasted for 5 months. The third refinement was done with 10 stages of aligners, with the objective of final detailing of the occlusion (Figures 3(f), 3(g), 3(h), 3(i), and 3(j)).

After three refinements, proper occlusion was finally achieved, orthodontic treatment was terminated and all attachments were removed. Vacuum-formed retainers were provided for retention. The patient was instructed on full-day retention for 1 year followed by nighttime retention for at least 1 year. The total treatment duration was 39 months, with excellent patient compliance.

**Figure 4:**
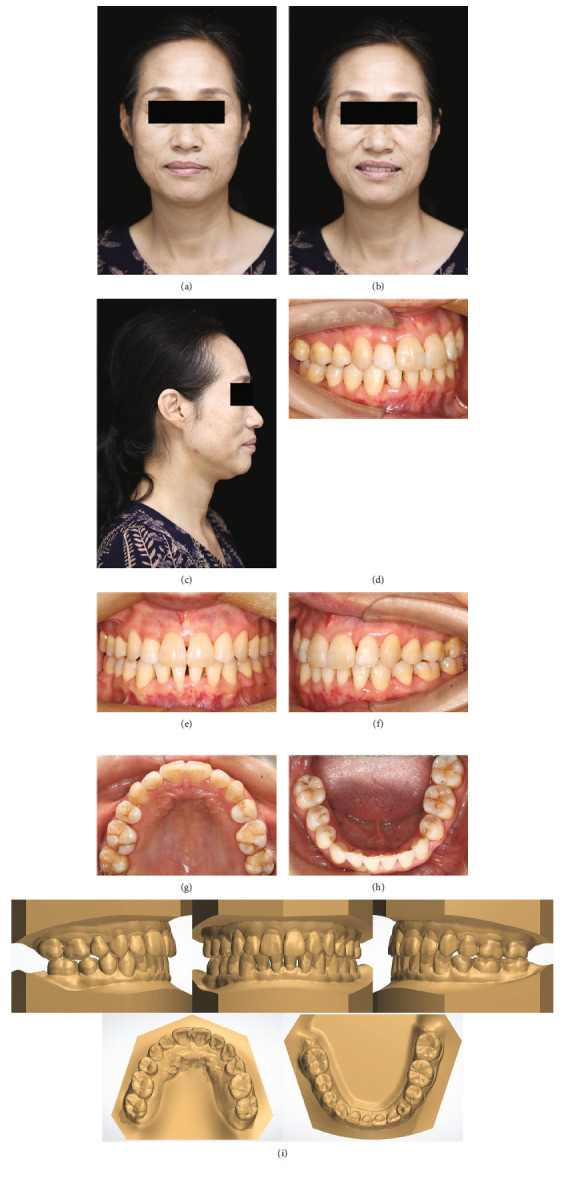
Posttreatment facial and intraoral photographs and dental casts. (a) Frontal view. (b) Smile. (c) Lateral view. (d) Right molar relationship. (e) Centric occlusion. (f) Left molar relationship. (g) Mirror view (maxillary). (h) Mirror view (mandibular). (i) Posttreatment dental casts.

**Figure 5:**
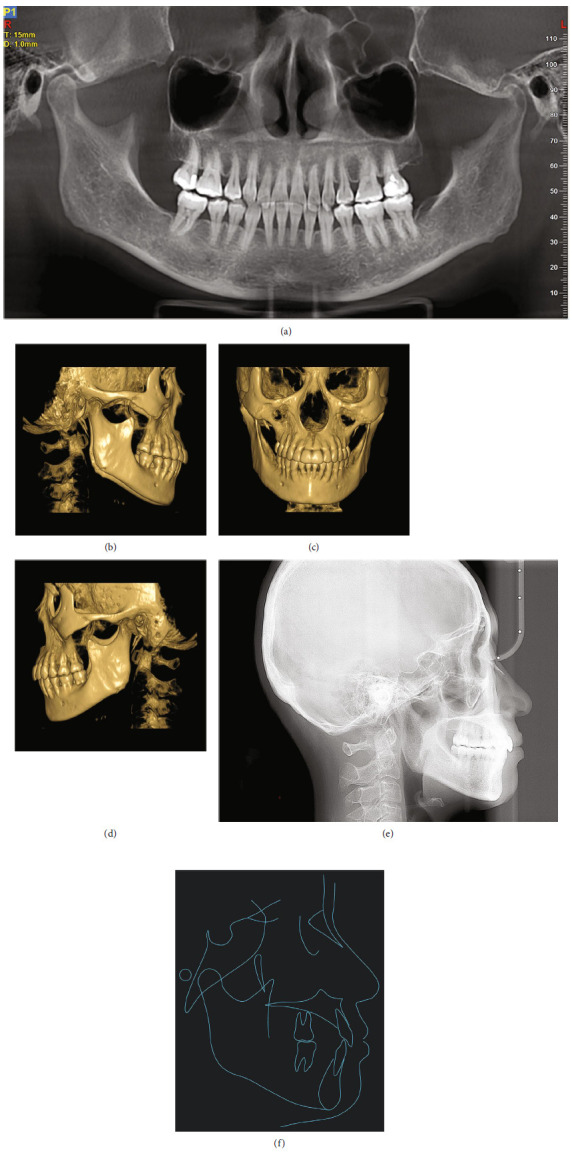
Posttreatment radiographs. (a) Panoramic radiograph sectioned from CBCT. (b–d) Three-dimensional reconstruction of CBCT. (e) Lateral cephalogram. (f) Lateral cephalometric tracing.

The posttreatment records (Figures 4 and 5; Table 1) demonstrated all the treatment objectives were accomplished. The intraoral photographs and dental casts (Figures 4(d), 4(e), 4(f), 4(g), 4(h), and 4(i))indicated satisfactory dental alignment, the harmonious relationship of dental arch widths, and symmetric arches, and the excessive overjet and overbite had been relieved. Coordinated intercuspal occlusal contact was achieved. Although the overjet and overbite were still slightly deeper than normal, considering the patient's periodontal status was not very fine, further vertical and torque control of the anterior teeth had not been performed. However, because of gingival recession caused by aging and periodontitis, and the triangular-shaped crown form, black triangular spaces between anterior teeth formed inevitably. To mitigate the esthetic damage caused by the black triangles, following the completion of the orthodontic treatment, several treatment approaches were recommended to the patient. The first option was periodontal plastic surgery, which involved gingival papilla reconstruction through soft tissue grafting to overcome unsightly black triangles. The second option was the tooth recontouring procedure, which included ceramic veneer or composite resin restorations to reshape the teeth. The third option was tissue volumising, which involved injecting tissue volumisers, such as hyaluronic acid, to augment the gingival papilla and reduce the black triangles in the esthetic zone. However, the patient reported no significant concerns regarding the gingival recession, and therefore, suggestions for further esthetic enhancement were declined. The facial photographs (Figures 4(a), 4(b), and 4(c)) revealed a more harmonious and balanced soft-tissue profile, with favorable incisor exposure during a smile, and the dentition midlines were aligned to the facial midline. No change in breathing or discomfort of the temporomandibular joint was reported after the orthodontic treatment, and the patient was satisfied with the results.

**Figure 6:**
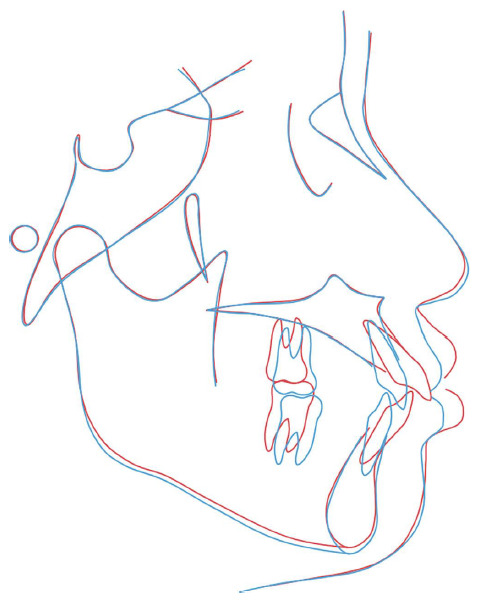
Superimpositions of the pretreatment (*red*) and posttreatment (*blue*) cephalometric tracings (on the S–N plane at the S point).

The posttreatment CBCT (Figures 5(a), 5(b), 5(c), and 5(d))demonstrated appropriate root parallelism and satisfactory space, with no substantial marginal bone loss or root resorption. The cephalometric analysis (Figures 5(e) and 5(f); Table 1) and superimposition (Figure 6) revealed that the protrusive incisors had been considerably retracted. The retraction and proper incisor position (Figures 5(b), 5(c), and 5(d)) contributed to an improved lip posture accordingly. The mandibular plane angle was maintained after the orthodontic treatment.

## 3. Discussion

In recent years, the proportion of adults among people seeking orthodontic treatment is increasing. It was reported that the ratio of adults in orthodontic patients grew from 15.4% to 21.0% between 1981 and 2017in the United States [5]. A survey made by the British Orthodontic Society reported that the number of adult orthodontic patients in private practice in 2018 was 5% more than that in 2016 [6]. The number of those who are middle-aged and above seeking orthodontic treatment is continuously increasing as well. An investigation pointed out that the ratio of middle-aged orthodontic patients in Asian countries with aging populations doubled between 2008 and 2012 [7].

It is widely approved that orthodontic treatment for middle-aged or elderly people is generally more challenging than that for adolescents or young adults due to various limitations [8]. This is partly because of the reality that the prevalence rate of chronic periodontal diseases in middle-aged patients is higher [9, 10]. On the other hand, middle-aged patients' surrounding period ontium is generally more hypoactive in responding to orthodontic force owing to the aging changes. However, mounting evidence has been documented proving that when the preexisting period ontitis is under control and good habits of oral hygiene are developed, a history of periodontal disease with alveolar bone loss is not a contraindication to orthodontic treatment [11, 12]. Furthermore, positive effects of periodontal treatment followed by orthodontic treatment on increasing levels of periodontal clinical attachment and improving surrounding marginal bone height have also been reported [13]. Although the initial periodontal status of middle-aged adults is generally unfavorable owing to greater loss of marginal bone, it has been reported that after orthodontic treatment, middle-aged adults presented periodontal changes and outcomes similar to those of young adults [8]. In this patient, due to the thin gingival biotype as well as the preexisting periodontal disease, the gradually exposed black triangle spaces between anterior teeth during orthodontic treatment had inevitably become an aesthetic defect. Nevertheless, no significant loss of marginal bone was observed by contrasting the posttreatment CBCT with the initial record. The non-professionals have relatively low aesthetic sensitivity to the anterior teeth, and the patient was very satisfied with the treatment results and refused surgical treatment options for gingival recession.

In orthodontic treatments, premolars are the most frequently extracted teeth for correcting malocclusions [14]. However, when a patient has teeth with poor prognosis other than premolars and extractions are ineluctable in correcting the malocclusion, the aforementioned teeth can be considered to be extracted other than healthy teeth [15]. This patient had frequently felt pain and discomfort in the region of the mandibular right first molar during mastication before the initial visit, and diagnoses of root resorption and furcation lesion of the mandibular right first molar were made. In addition, a diagnosis of caries in her maxillary left second premolar was made. After a comprehensive assessment of the patient's condition, extractions of the maxillary right first premolar and left second premolar, and mandibular right first molar and left first premolar were determined to correct the malocclusion.

Adults often reject the use of fixed labial orthodontic appliances because of esthetic impairment. Clear aligners emerge as the times require. Clear aligners have currently been used for more complex orthodontic tooth movements, including tooth rotation, molar distalization, and dental expansion with the advancement of attachments and materials, and their application scope has been extended from non-extraction to extraction cases [4, 16–18]. Therefore, according to the requirement of this patient, we chose clear aligners to overcome the aesthetic defects in the orthodontic treatment process. Due to the poor prognosis of the mandibular right first molar and maxillary left second premolar, a treatment plan of asymmetric extraction was indicated for this patient. Because asymmetric extraction produced different amounts of space in each quadrant, more accurate control of anchorage was required in designing the plan of clear aligner treatment. In the maxillary dentition, because the extraction sites were the right first premolar and left second premolar, our design of tooth movements was to first distalize the left first premolar to the final position, and then start the aligning and retraction processes of anterior teeth. In the mandibular dentition, the right premolars were firstly distalized to the target position, then anterior teeth began to be aligned and retracted, and at the same time, mesial movement of the right second molar got started. From the perspective of the treatment results, our designs of anchorage control and sequence of tooth movements achieved ideal effects. Without skeletal anchorage devices, the processes of dentition aligning and space closure were completed, and no obvious loss of anchorage was observed. However, it is well known that the virtual simulation of clear aligner treatment is more a design of force application than modeling the final tooth position. Therefore, the actual tooth movements achieved after clear aligner treatment differs from those planned by the virtual setup. Even by using traditional fixed appliances, the treatment of a case with first molar extraction confronts great difficulties, and it is more challenging by using clear aligners. In this patient, roller-coaster effects including anterior interference, and posterior open bite occurred in the process of space closure, even if we had designed the control of anterior teeth torque and antitipping of posterior teeth. Research studies have demonstrated that the tooth movements designed in a virtual simulation of clear aligner treatment cannot be fully accomplished, ranging from 28% to 88% of the planned depending on the modes of tooth movements and the tooth types [18–21]. Thus, additional clear aligners (refinements) aiming at torqueing and intruding anterior teeth, and uprighting posterior teeth were prescribed to this patient. From the perspective of biomechanics, the retraction force (on anterior teeth) and protraction force (on posterior teeth) exerted by clear aligners are applied on crowns and pass through the occlusal side of the center of resistances, leading to distal tipping of canines, mesial tipping of posterior teeth, and lingual tipping and extrusion of incisors. Therefore, for extraction cases, additional control of anterior teeth torque and antitipping of teeth adjacent to the extraction space should be designed to increase the expression rate of expected tooth movements [22]. More specifically, to prevent unwanted tooth movements in extraction patients, designs of distal crown tipping of posterior teeth and mesial crown tipping of canines are necessary during space closure in clear aligner treatment [4].

## 4. Conclusion

Clear aligner treatment is a novel strategy to treat cases with asymmetric tooth extraction, and even if applied in middle-aged patients or molar extraction cases, its treatment effects are still reliable.

## Figures and Tables

**Table 1:** Cephalometric measurements.

Measurement	Normal	Pretreatment	Posttreatment
SNA (°)	83.0 ± 4.0	86.7	86.7
SNB (°)	80.0 ± 3.0	82.2	82.2
ANB (°)	3.0 ± 2.0	4.5	4.5
FMIA (°)	54.9 ± 6.1	48.1	68.8
FMA (°)	31.3 ± 5.0	27.2	27.1
IMPA (°)	93.9 ± 6.2	104.8	84.0
MP-SN (°)	33.0 ± 4.0	34.4	34.6
PP-GoGn (°)	21.0 ± 4.0	24.6	24.6
Go-Pog (mm)	73.0 ± 4.0	70.3	71.1
Go-Co (mm)	56.0 ± 4.0	51.7	53.5
N-ANS (mm)	53.0 ± 3.0	51.3	51.2
S-Go (mm)	75.0 ± 5.0	70.3	71.9
S-Go/N-Me (%)	66.0 ± 4.0	63.3	63.7
ANS-Me/N-Me (%)	53.0 ± 2.0	53.9	54.6
U1-SN (°)	105.0 ± 6.0	116.9	97.9
U1-NA (°)	21.0 ± 6.0	30.2	11.2
U1-NA (mm)	4.0 ± 2.0	8.8	1.7
U1-APo (mm)	7.0 ± 2.0	13.0	5.9
U1-PP (mm)	28.0 ± 2.0	25.7	27.5
U1-L1 (°)	127.0 ± 9.0	103.9	143.5
L1-NB (°)	28.0 ± 6.0	41.4	20.8
L1-NB (mm)	6.0 ± 2.0	10.7	4.3
L1-APo (mm)	3.0 ± 2.0	8.5	1.8
L1-MP (mm)	40.0 ± 2.0	41.4	40.2
Wits (mm)	0.0 ± 2.0	−3.2	−1.5
UL-EP (mm)	2.0 ± 2.0	2.0	−2.0
LL-EP (mm)	3.0 ± 2.0	6.3	0.9

## Data Availability

Data supporting this research article are available from the corresponding author or first author on reasonable request.

## Abbreviation

CBCT: Cone beam computed tomography.
